# Duodenal gastrointestinal stromal tumor resembling a pancreatic neuroendocrine tumor in a patient with neurofibromatosis type I (von Recklinghausen's disease): a case report

**DOI:** 10.1186/1752-1947-4-302

**Published:** 2010-09-08

**Authors:** Shinji Ohtake, Noritoshi Kobayashi, Shingo Kato, Kensuke Kubota, Itaru Endo, Yoshiaki Inayama, Atsushi Nakajima

**Affiliations:** 1 Gastroenterology Division, Yokohama City University Hospital, 3-9-Fukuura, Kanazawa-ku Yokohama 236-0004, Japan; 2 Gastroenterological Surgery Division, Yokohama City University Hospital, Yokohama, Japan; 3 Pathological Division, Yokohama City University Hospital Yokohama, Japan

## Abstract

**Introduction:**

Gastrointestinal stromal tumor is the most frequent nonepithelial tumor found in the gastrointestinal tract. One important clinical problem is that gastrointestinal stromal tumors, especially the extramural growth type, can be difficult to distinguish from other organ tumors. The case of a patient with an extramural gastrointestinal stromal tumor of the duodenum that mimicked a pancreatic head tumor has previously been reported. Here, we report a rare case of a patient with a duodenal gastrointestinal stromal tumor with extramural growth that mimicked a pancreatic neuroendocrine tumor. In this case, the gastrointestinal stromal tumor was also associated with neurofibromatosis type 1 (also known as von Recklinghausen's disease). To the best of our knowledge, this is the first report to describe the case of a patient with a duodenal gastrointestinal stromal tumor with neurofibromatosis type 1 in which the radiological findings resembled those of a pancreatic neuroendocrine tumor.

**Case presentation:**

A 60-year-old Japanese woman with a history of neurofibromatosis type 1 was admitted to our hospital for the treatment of a tumor of her pancreas. She had no symptoms, but an abdominal ultrasonography screening examination had revealed a hypoechoic mass in the head of her pancreas. Laboratory data, including tumor markers, were within the normal ranges, and her insulin and glucagon levels were also within the normal ranges. However, her plasma gastrin level was elevated at 580 pg/mL (30 to 150 pg/mL). A computed tomography examination revealed a hypervascular tumor measuring 14 mm in diameter in the head of her pancreas. We diagnosed the patient as having a pancreatic neuroendocrine tumor and performed a tumor resection with a duodenal wedge resection. Microscopic analysis revealed spindle cell tumors in a trabecular pattern. The patient was finally diagnosed as having a duodenal gastrointestinal stromal tumor of the uncommitted type.

**Conclusion:**

Extramural growth-type gastrointestinal stromal tumors can be difficult to distinguish from other organ tumors. In our case, a duodenal gastrointestinal stromal tumor was difficult to distinguish from a pancreatic neuroendocrine tumor based on radiological findings. When patients are identified as having hypervascular lesions that have adhered to the gastrointestinal tract, the possibility of an extramural growth-type gastrointestinal stromal tumor as a differential diagnosis should be considered in patients with neurofibromatosis type 1.

## Introduction

Gastrointestinal stromal tumor (GIST) is the nonepithelial tumor that occurs most frequently in the gastrointestinal tract. One important clinical problem is that GIST, in particular the extramural growth type, can be difficult to distinguish from other organ tumors. In particular, duodenal extramural GIST is difficult to distinguish from a pancreatic tumor. In 2005, Uchida *et al. *first reported the case of a patient with an extramural GIST of the duodenum that had mimicked a tumor of the pancreatic head [1]. Here, we report a rare case of a patient with duodenal extramural growth-type GIST that was associated with neurofibromatosis type 1 (NF1; also known as von Recklinghausen's disease). NF1-associated gastrointestinal lesions include not only GIST, but also hyperplastic lesions of intestinal neural tissue and its supporting structures and endocrine cell tumors of the duodenum and periampullary regions [2]. Our case described here was rare and difficult to distinguish from a pancreatic neuroendocrine tumor based on radiological findings and etiological features.

## Case presentation

**Figure 1:**
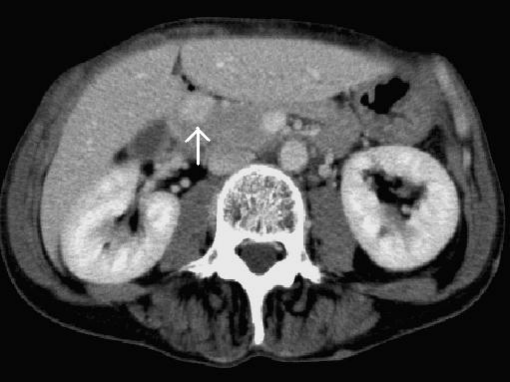
**An enhanced computed tomography scan revealing a hypervascular tumor in the head of the patient's pancreas (arrow)**.

A 60-year-old Japanese woman with a history of rheumatoid arthritis and NF1 was admitted to our hospital for the treatment of a tumor of a pancreas. She had no symptoms, but an abdominal ultrasonography screening examination had revealed a hypoechoic mass in the head of a pancreas. Laboratory data, including measurement of tumor markers, were within the normal ranges, and her insulin and glucagon levels were also within the normal ranges. However, the plasma gastrin level was elevated at 580 pg/mL (30 to 150 pg/mL). A computed tomography (CT) examination revealed a hypervascular tumor measuring 14 mm in diameter in the pancreas head (Figure 1). Magnetic resonance imaging (MRI) also revealed a massive tumor in the head of the pancreas. A duodenal endoscopy revealed that the lumen of the patient's duodenum was not compressed by the extraluminal tumor, and findings on endoscopic retrograde cholangiopancreatography showed that her main pancreatic duct was not stenosed or blocked. We diagnosed the patient as having a pancreatic neuroendocrine tumor and planned to perform a tumoral enucleation from her pancreas.

A whitish elastic hard nodule was easily removed from the pancreatic parenchyma, but the tumor was connected to her duodenal wall via a stalk. Consequently, we performed a tumor resection with a duodenal wedge resection. As a small whitish nodule was also found in the patient's jejunum during the initial operation, a segmental jejunectomy was also performed. A histological examination of frozen sections of the patient's tumors revealed spindle cells with connective tissues. Thus, we diagnosed the patient as having multiple GISTs and did not perform a more radical resection.

**Figure 2:**
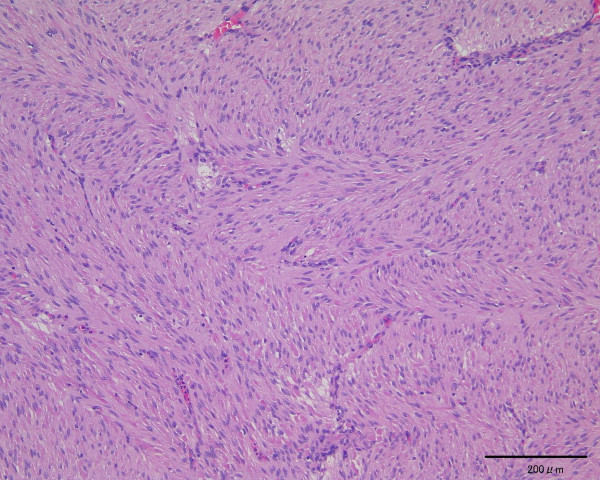
**Microscopic appearance of the tumor**. Stained with hematoxylin and eosin. Magnification × 100.

Macroscopically, the resected specimens consisted of solid and hard masses that were connected to the patient's duodenal and jejunal walls but not to the parenchyma of the head of her pancreas. Microscopically, this neoplasm originated from the muscularis propria of the duodenum wall and consisted of spindle cells in a trabecular pattern without necrosis (Figure 2). Less than five mitoses per 50 high-power fields were observed.

**Figure 3:**
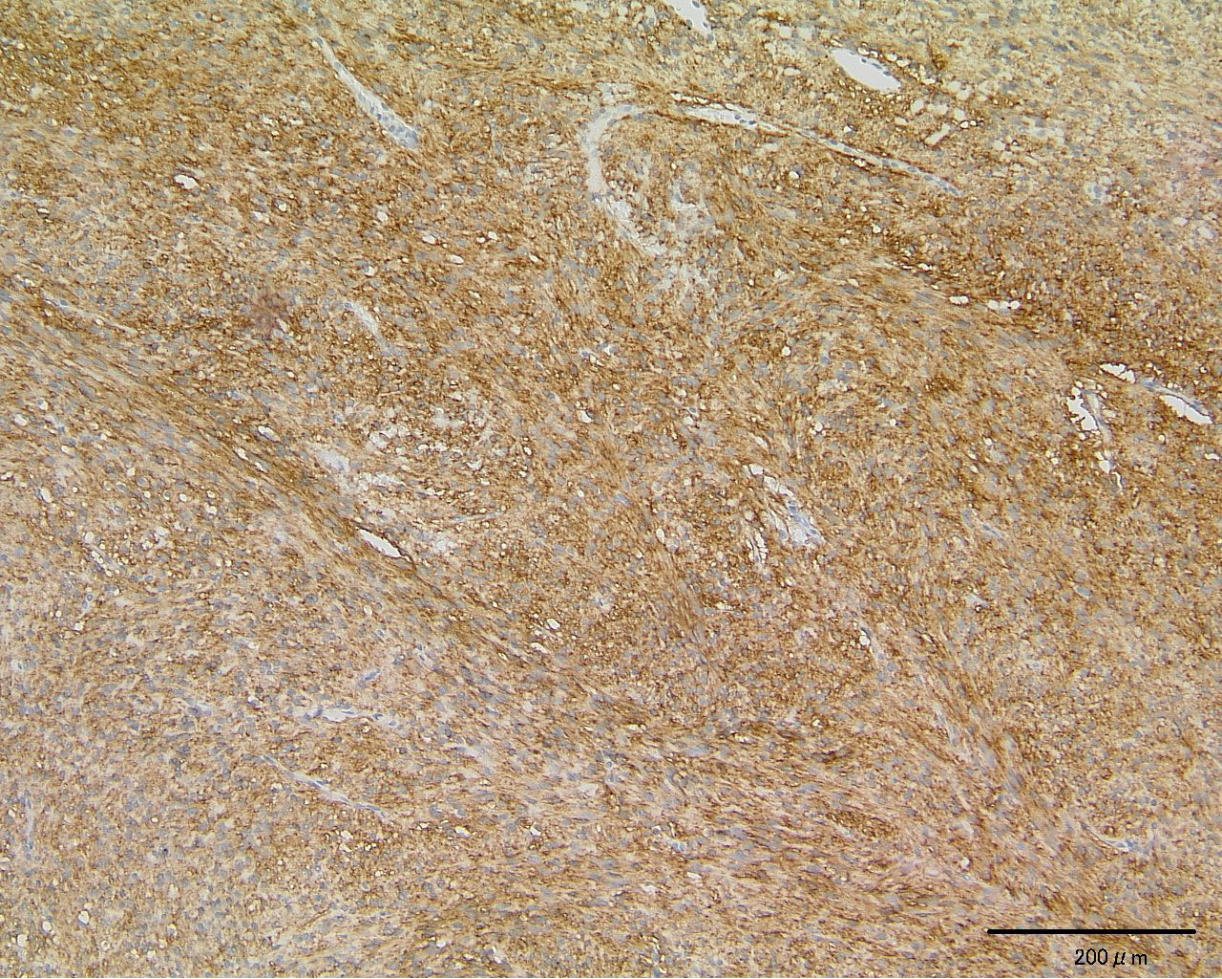
**Microscopic appearance of the tumor**. c-kit immunohistochemistry. Magnification × 100.

On immunohistochemistry, both of the patient's tumors were diffusely positive for the type III receptor tyrosine kinase (TK) named KIT (Dako Cytomation, Copenhagen, Denmark; Figure 3), whereas her duodenal tumor was focally positive and her jejunal tumor was diffusely positive for CD34 (Nichirei, Tokyo, Japan). Both of the patient's tumors were negative for smooth muscle actin (Dako Cytomation, Copenhagen, Denmark) and S-100 (Nichirei, Tokyo, Japan). We finally diagnosed the patient as having duodenal and jejunal GISTs of uncommitted type. Patients with these GISTs are regarded as being very low risk of recurrence according to the National Comprehensive Cancer Network (NCCN) guidelines [3]. The patient has remained healthy for two years without any recurrences after surgery.

## Discussion

GISTs are the most frequently occurring nonepithelial tumor of the gastrointestinal tract. Based on the recent discovery that GIST and the intestinal cell of Cajal (ICC) express CD34 and KIT, GISTs are now considered to develop from ICC or to differentiate into ICC. Most GISTs carry mutations in the proto-oncogene *c-kit*, which constitutively activates KIT kinase when translated; this gain-of-function mutation in the *c-kit *gene is considered to be the cause of GISTs [4]. After the stomach, the small intestine is the second most common primary site for GISTs. Miettinen *et al. *[5] reported that duodenal GISTs most frequently involve the second portion of the duodenum, followed by the third, fourth, and first portions. They also reported that many tumors are comprised of a gross ulceration of the mucosa, with a component that bulged underneath the mucosa, forming a partly intramural mass with a centrally ulcerated umbilication.

In our case report, the patient's tumor exhibited only extramural growth, and no specific intramural change in the duodenum was present. These clinical features are rare [5], so it was difficult for us to distinguish a GIST from a tumor of the pancreas. Therefore, we first considered this tumor to be a neuroendocrine tumor in the head of the patient's pancreas, based on the tumors radiological and duodenal endoscopic findings [6]. CT studies indicated that GISTs are hypervascular and may have cystic and necrotic components combined with intramural and extramural tumor growth and signs of malignancy. Small tumors are depicted on CT scans as sharply marginated smooth masses with moderate contrast enhancement [7]. In our case report, the patient's radiological findings were typical of a GIST, but the extramural growth made it difficult to distinguish the GIST from other types of tumors, especially a pancreatic neuroendocrine tumor. Furthermore, the patient's plasma gastrin level was slightly elevated. Consequently, we misdiagnosed this tumor as a gastrinoma. However, the immunohistochemical features of both tumors (duodenum and jejunum) were diffusely negative for gastrin. Furthermore, almost all gastrinomas are located in the "gastrinoma triangle". We carefully examined this area, but no other tumors were observed. After tumor resection, the plasma gastrin level did not change. The plasma gastrin level might have been slightly elevated in this case as a result of atrophic gastritis. In fact, an upper endoscopy and biopsy specimen revealed severe atrophic gastritis. Because of the absence of acid inhibition, the functional G cells were stimulated and gastrin secretion was increased [8].

NF1 is caused by a mutation of the *NF1 *gene, but the mutations are heterogeneous and the diagnosis of NF1 is still based largely on clinical criteria. Many patients with NF1 will have a GIST, because it occurs in approximately 11 to 25% of all NF1 patients [9]. Sporadic GISTs are most commonly found in the stomach and contain mutated KIT or plate-derived growth factor receptor (PDGFR) protein; point mutations in the *c-kit *or *PDGFR *genes have also been identified. However, NF1 that is associated with GIST is rarely associated with these other mutations, and multiple GISTs are commonly found in the small intestine. The histologic and immunohistochemical differences between GISTs in NF1 patients and non-NF1 patients have not been fully clarified.

According to the NCCN guidelines [3], the GISTs in our case were associated with a very low risk of recurrence and the patient should be followed up every six months with a CT scan. No obvious evidence exists that adjuvant chemotherapy for resected GISTs might prolong the post-operative survival times of patients. Thus, our case was not treated with adjuvant imatinib therapy but was followed up every six months with CT for two years. Fortunately, no new tumors were observed in this time.

The most important issue in surgical strategies for patients with GIST is whether a complete resection can be achieved. A variety of surgical methods can be used to obtain a complete resection, such as duodenopancreatectomy, duodenal wedge resection, and segmental duodenal resection. However, in this very low-risk group of completely resectable GISTs, tumor resection with a duodenal wedge resection and segmental jejunectomy might be suitable surgical methods.

NF1-associated gastrointestinal lesions include not only GISTs, but also hyperplastic lesions of intestinal neural tissue and its supporting structures, and endocrine cell tumors of the duodenum and periampullary region [2]. In our case, a duodenal GIST was difficult to distinguish from a periampullary endocrine tumor based not only on the radiological findings, but also the etiological features.

## Conclusions

A rare and difficult case of duodenal extramural GIST associated with NF1 is presented. Duodenal extramural growth GISTs are difficult to distinguish from pancreatic neuroendocrine tumors, especially in patients with NF1.

## Abbreviations

CT: computer tomography; GIST: gastrointestinal stromal tumor; ICC: intestinal cell of Cajal; MRI: magnetic resonance imaging; NCCN: National Comprehensive Cancer Network; NF1: neurofibromatosis type 1.

## Consent

Written informed consent was obtained from the patient for publication of this case report and any accompanying images. A copy of the written consent is available for review by the Editor-in-Chief of this journal.

## Competing interests

The authors declare that they have no competing interests.

## Author's contributions

SO drafted the article. NK was involved in the conception and design of the study and critical revision of the article for important intellectual content, and gave final approval for publication of the article. SK was involved in the conception and design of the study and patient care. KK was involved in the conception and design of the study and patient care. IE was involved in the conception and design of the study and patient care. YI was involved in the conception and design of the study and patient care. AN gave final approval for publication of the article. All authors have read and approved the final manuscript.
